# Delay-Doppler Domain Time-Hopping Key Generation and Security Analysis for Orthogonal Time Frequency Space Satellite Communication Systems

**DOI:** 10.3390/s26103230

**Published:** 2026-05-20

**Authors:** Wei Li, Zhendie Bai, Jikang Wang, Xiaofan Xu, Xianggeng Zhu

**Affiliations:** 1 Shanghai Satellite Network Research Institute Co., Ltd., Shanghai 201210, China; liwei.nudt.cn@gmail.com (W.L.); jikangwang_2019@163.com (J.W.); zhuk317@sina.com (X.Z.); 2 Shanghai Key Laboratory of Satellite Network, Shanghai 201210, China; 3 State Key Laboratory of Satellite Network, Shanghai 201210, China

**Keywords:** key generation, satellite communication, OTFS, delay-doppler domain, time-hopping mechanism, channel characteristics

## Abstract

Physical-layer key generation (PLKG) is a technique that produces symmetric encryption keys by exploiting the inherent characteristics of wireless channels. It offers advantages including high physical-layer security, elimination of pre-shared keys, dynamic upgradability, and resistance to quantum attacks, making PLKG a promising security solution for next-generation (6G) networks. However, satellite communication channels exhibit high dynamics and long propagation delays. Characteristics such as large Doppler shifts, short coherence times, and orbital predictability pose severe challenges to PLKG, including reciprocity degradation, low key generation rate (KGR), and susceptibility to channel-prediction attacks. This work proposes a delay-Doppler domain time-hopping key generation scheme (KE-DD-TH) based on Orthogonal Time Frequency Space (OTFS) modulation for high-speed links between Low-Earth-Orbit (LEO)/Medium-Earth-Orbit (MEO) satellites and ground terminals in Ka/Ku bands. The scheme performs non-uniform sampling on the DD domain grid of OTFS symbols using an ephemeris-driven pseudo-random time-hopping sequence generated by cascaded linear feedback shift registers (LFSRs) and a nonlinear matrix transformation. Both legitimate parties estimate the channel only at time-hopping instants and multiply two adjacent estimates to construct an “equivalent channel” matrix, yielding a random source with high entropy, high reciprocity, and low predictability. The eavesdropper’s key disagreement rate (KDR) remains close to 0.5 under all signal-to-noise ratio (SNR) conditions, corresponding to the ideal random-guessing baseline. This indicates that Eve obtains negligible mutual information, i.e., $I(K_{A};K_{E})\approx 0$. By contrast, the conventional KE-DD scheme allows Eve’s KDR to degrade to 0.014 at 30 dB SNR, indicating near-complete key recovery. The generated keys pass all 12 randomness tests of the NIST SP 800-22 statistical test suite.

## 1. Introduction

With the rapid deployment of global satellite internet constellations, satellite-to-ground wireless communication has become a critical infrastructure supporting coverage in remote areas, emergency communications, and the global Internet of Things (IoT). Satellite internet scenarios exhibit distinctive characteristics, including wide-area coverage, high-speed mobility (LEO satellite orbital velocity is approximately 7.2 km/s), long propagation delays (Geostationary Earth Orbit (GEO) satellites incur about 250 ms), and complex Doppler effects (LEO Doppler shift up to ±50 kHz), imposing stringent requirements on physical-layer security. Compared to terrestrial mobile communication channels, satellite communication channels demonstrate markedly different physical properties, posing severe challenges to conventional physical-layer key generation (PLKG) techniques, as summarized in Table 1.

Although the high-speed mobility of satellites theoretically provides richer channel randomness, the aforementioned features lead to bottlenecks in conventional schemes (e.g., orthogonal frequency division multiplexing (OFDM)-based PLKG) under satellite scenarios, including key agreement failure, limited KGR, and susceptibility to channel-prediction attacks [1,2,3,4].

Related work on OTFS-based security and satellite PLKG: OTFS modulation, through DD domain signal processing, has recently attracted considerable attention for doubly dispersive channels [5,6,7,8]. Gaudio et al. [5] demonstrated that OTFS outperforms OFDM in sparse channels, while Xiao et al. [6] provided a comprehensive overview of OTFS for IoT applications. On the physical-layer security side, Gunjan et al. [8] analyzed the secrecy capacity of OTFS systems under passive eavesdropping and transmit antenna selection, establishing the physical-layer security advantage of OTFS in high-mobility scenarios. For MIMO-OTFS channels specifically, Bora et al. [9] analyzed spatial correlation effects that must be mitigated for reliable channel estimation. In the satellite PLKG domain, Hao et al. [10] proposed a multi-satellite cooperation scheme with random perturbation for LEO satellite-to-ground key generation, which improves randomness but does not address the channel prediction vulnerability introduced by public orbital mechanics. Despite this progress, no existing work combines OTFS DD domain channel features with a time-hopping mechanism specifically designed for satellite PLKG, leaving the joint problem of prediction resistance, low KGR, and reciprocity degradation unsolved.

Threat model: With the integrated application of reconfigurable intelligent surfaces (RIS), deep-learning-aided channel prediction [11], and integrated sensing and communication (ISAC) [12,13,14], eavesdropping threats are becoming increasingly sophisticated. Eavesdroppers can leverage deep neural networks to predict satellite channel states from publicly available ephemeris data, actively manipulate RIS phase configurations to disrupt key agreement, or exploit ISAC sensing capabilities to precisely locate and track legitimate communicating parties.

Proposed scheme: To address these challenges, this work proposes an OTFS KE-DD-TH scheme with the following mechanisms:

- Time-hopping mechanism: Uses ephemeris-driven pseudorandom sequences for non-uniform sampling on delay-Doppler (DD) domain grid points;
- Equivalent channel construction: Forms the product of two adjacent channel estimates to enhance source entropy and resist prediction attacks;
- Three-dimensional random source: Integrates “ephemeris–channel–time” dynamic factors to counter prediction attacks.

Contributions: The key contributions of this paper, each framed against prior work, are as follows:

1. First OTFS DD domain PLKG scheme for LEO/MEO satellites: Unlike Hao et al. [10] (which uses orbital diversity but uniform sampling and is vulnerable to AI-assisted prediction) and Gunjan et al. [8] (which analyzes secrecy capacity but does not generate keys), we propose the first key generation protocol that exploits the sparse DD domain structure of satellite channels for key extraction.
2. Ephemeris-driven time-hopping with provable unpredictability: The non-uniform sampling instants are generated by a cascaded nonlinear LFSR with period 3937, making the equivalent channel $\hat{H}\left(t_{i}\right)\hat{H}\left(t_{i+1}\right)$ statistically independent of any single-instant prediction achievable by Eve, even with full ephemeris knowledge. We formally bound Eve’s mutual information as $I(K_{A};K_{E})\approx 0$.
3. Formally modeled attack scenarios: Unlike prior satellite PLKG works, we provide explicit mathematical models for AI prediction, RIS manipulation, and CubeSat close-proximity eavesdropping attacks, and demonstrate security against all three.

## 2. System Model and Proposed Scheme

### 2.1. System Model

**Figure 1:**
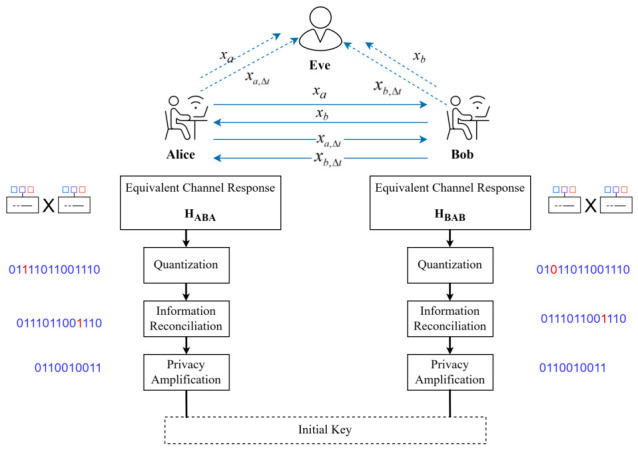
Time-Hopping-Based Delay-Doppler Domain Key Extraction.

To counter prediction attacks, RIS manipulation, and close-proximity eavesdropping in satellite internet, the key randomness source must extend beyond single-channel randomness to a three-dimensional joint randomness source integrating ephemeris, channel, and time. This work proposes a satellite-to-ground time-hopping key generation architecture based on the DD domain. The system model is illustrated in Figure 1:

- Alice: A LEO satellite equipped with a single antenna and operating in digital transparent forwarding (bent-pipe) mode, transmitting OTFS pilot signals via Ka/Ku feeder links or inter-satellite links (ISLs).
- Bob: A ground user terminal (UT) or another LEO satellite; if Bob is a ground terminal, the link is a typical satellite-to-ground TDD; if Bob is also a satellite, the link is an inter-satellite TDD.
- Eve: A composite eavesdropping network.

Alice and Bob, as legitimate communicating parties, need to generate symmetric keys from satellite-to-ground wireless channel state information. Eve is a passive eavesdropper that can intercept transmitted signals between Alice and Bob and attempt to extract the generated keys. With the continuous advancement of eavesdropping technologies and equipment, Eve’s methods have become increasingly sophisticated, including the following:

- Ground-based large-aperture phased array + AI server: Leverages publicly available ephemeris, real-time Global Navigation Satellite System (GNSS) corrections, and atmospheric models to achieve millimeter-level Doppler-shift and sub-millisecond delay prediction of the satellite-to-ground channel [10,15].
- Maneuverable picosatellite/CubeSat: (mass ≤ 10 kg, Δv≥ 50 m/s): Capable of maneuvering within minutes to within half-wavelength (λ/2 ≈ 1–2 cm at Ka-band) of the Alice–Bob link for close-proximity eavesdropping [15].
- Reconfigurable intelligent surface foil (RIS-Foil) or Micro-RIS-SAT: Deploys reflective arrays on or near satellites to dynamically adjust phase and amplitude in real time, reconstructing legitimate channel characteristics and disrupting key agreement [16,17,18].

For analytical simplicity, a single-antenna system is considered, with communication operating in Time Division Duplex (TDD) mode.

### 2.2. Improved M-Sequence-Based Random Time Interval Generation Using Time-Hopping Sequences

**Figure 2:**
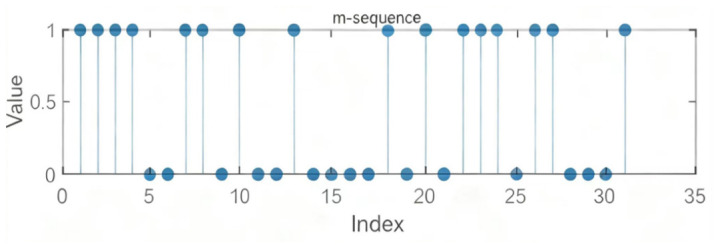
The 5th-Order M-Sequence with Feedback Polynomial.

Common pseudorandom sequences for time-hopping include M-sequences, Gold sequences, and Kasami sequences. Among them, the M-sequence is a pseudorandom sequence generated by a linear feedback shift register (LFSR). We utilize M-sequences to generate random time intervals. Figure 2 illustrates a 5th-order M-sequence with feedback polynomial $f\left(x\right)=x^{5}+x^{2}+1$, which exhibits no repeated pattern within one full period, providing long-term unpredictability. To mitigate the predictability risk inherent in linear feedback and enhance security, we employ cascaded multi-stage LFSRs to significantly extend the sequence period. For example, combining a 5th-order and a 7th-order LFSR yields a period of up to $\left(2^{5}-1\right)\cdot \left(2^{7}-1\right)=3937$. Furthermore, through nonlinear combination and forward feedback matrix transformation, the linear complexity is substantially increased, approaching or exceeding the product of the individual LFSR orders. This enhances the sequence’s spreading performance and security in communication and encryption applications, effectively resisting linear attacks such as the Berlekamp–Massey algorithm.

#### 2.2.1. Multi-Stage LFSR Cascading

A 5th-order LFSR (feedback polynomial $f_{1}\left(x\right)=x^{5}+x^{2}+1$, initial state [1, 1, 1, 1, 0], period 31) and a 7th-order LFSR (feedback polynomial $f_{2}\left(x\right)=x^{7}+x^{6}+1$, initial state [1, 0, 0, 0, 0, 0, 0], period 127) are employed. The two sequences are combined via a nonlinear Boolean function $g\left(x_{5},x_{6},x_{7}\right)=\left(x_{5}\cdot x_{7}+x_{6}\right)\;\mathrm{mod}\;2$, yielding a composite sequence with period 31×127=3937. This Boolean function introduces nonlinear product terms $x_{5}\cdot x_{7}$ and combines the bit $x_{6}$ of the 7th-order LFSR, to ensure output balance (equal probability of 0 and 1) and high nonlinearity, thereby enhancing the statistical randomness and attack resistance of the sequence.

#### 2.2.2. Forward Feedback Matrix Transformation

After passing through the nonlinear combination module, an enhanced M-sequence ($M_{\mathrm{enh}}$) is generated. The $M_{\mathrm{enh}}$ sequence is then segmented into sub-sequences $m^{\prime }\left(i\right)$ of length *k* ($1\leq k\leq 2^{n}-2$); the last sub-sequence, if of length less than *k*, is zero-padded. Let *i* denote the index of the sub-sequence, representing the *i*-th segmented sub-sequence. The numerical value of each sub-sequence is $|m^{\prime }\left(i\right)|$, and the time granularity is defined as the coherence time $\bar{t}$. The resulting random time interval is given by

(1) $$\left\{\Delta t\right\}=\left\{(2^{k}-|m^{\prime }\left(i\right)|+|m^{\prime }\left(i+1\right)\left|\right)\bar{t}\right\},\;1\leq i\leq \left\lceil \frac{2^{n}-1}{k}\right\rceil -1$$

where ⌈·⌉ denotes the ceiling function. The nonlinear enhancement improves sequence randomness, yielding a more uniform distribution of time intervals and reduced risk of pattern repetition.

**Figure 3:**
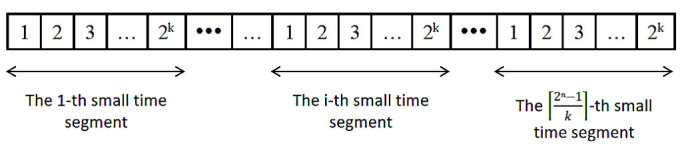
Schematic of Channel Sounding Time Division.

Figure 3 illustrates the time-division scheme, where the total duration *t* required for channel sounding is segmented at intervals of $\bar{t}$. It is assumed that each signal transmission is completed within a single $\bar{t}$ duration grid. Using the proposed random time interval generation method, the length of each segmented small time interval is $2^{k}\bar{t}$. Due to zero-padding of the last sub-sequence when its length is insufficient during segmentation, the total number of resulting small time segments is $\left\lceil \left(2^{n}-1\right)/k\right\rceil$. Therefore, the following relationship holds:

(2) $$t=\left\lceil \frac{2^{n}-1}{k}\right\rceil 2^{k}\bar{t}$$

After the nonlinear sequence enhancement, the computational formula for *t* remains unchanged; however, the unpredictability of the sequence is significantly improved, thereby enhancing channel security.

#### 2.2.3. Random Time Interval Generation

Assume the enhanced M-sequence ($M_{\mathrm{enh}}$) is segmented into sub-sequences of length 4. Using the 7th-order LFSR combined with the forward feedback matrix, the generated enhanced sequence is

$$M_{\mathrm{enh}}=\left[1000011100010100010101111011111\right]$$

Then segmented into sub-sequences of length 4, we obtain 1000, 0111, 0001, 0100, 0100, 0111, 1011, 1011. Each sub-sequence is interpreted as a binary vector $V=[v_{1},v_{2},v_{3},v_{4}]$. A 4 × 4 binary transformation matrix *W* is applied:

(3) $$W=\left[\begin{array}{cccc} 1 & 0 & 1 & 1\\ 0 & 1 & 1 & 0\\ 1 & 1 & 0 & 1\\ 0 & 1 & 1 & 1 \end{array}\right]$$

to compute the transformed sub-sequences $V^{\prime }=\left(V\cdot W\right)\;\mathrm{mod}\;2$. The resulting time interval set is $\left\{13\bar{t},11\bar{t},19\bar{t},16\bar{t},18\bar{t},12\bar{t},16\bar{t}\right\}$.

The enhanced $M_{\mathrm{enh}}$ sequence significantly outperforms the standard M-sequence in randomness and security, and is widely applicable in spread-spectrum communications, pseudorandom number generation, signal synchronization, spectrum analysis, and cryptographic applications.

### 2.3. DD Domain Key Extraction Based on Random Time Intervals

Let the signal transmitted by Alice be

(4) $$x={\left[x\left[0,0\right],\cdots x\left[N-1,0\right],x\left[0,1\right],\cdots x\left[N-1,1\right],\cdots x\left[N-1,M-1\right]\right]}^{T}$$

Let the signal received by Bob be

(5) $$y={\left[y\left[0,0\right],\cdots y\left[N-1,0\right],y\left[0,1\right],\cdots y\left[N-1,1\right],\cdots y\left[N-1,M-1\right]\right]}^{T}$$

Let the channel noise be

(6) $$z={\left[z\left[0,0\right],\cdots z\left[N-1,0\right],z\left[0,1\right],\cdots z\left[N-1,1\right],\cdots z\left[N-1,M-1\right]\right]}^{T}$$

and the channel H be a vectorized symbol in the DD domain, satisfying $x,y,z\in C^{NM\times 1}$ and $H\in C^{NM\times NM}$.

**Figure 4:**
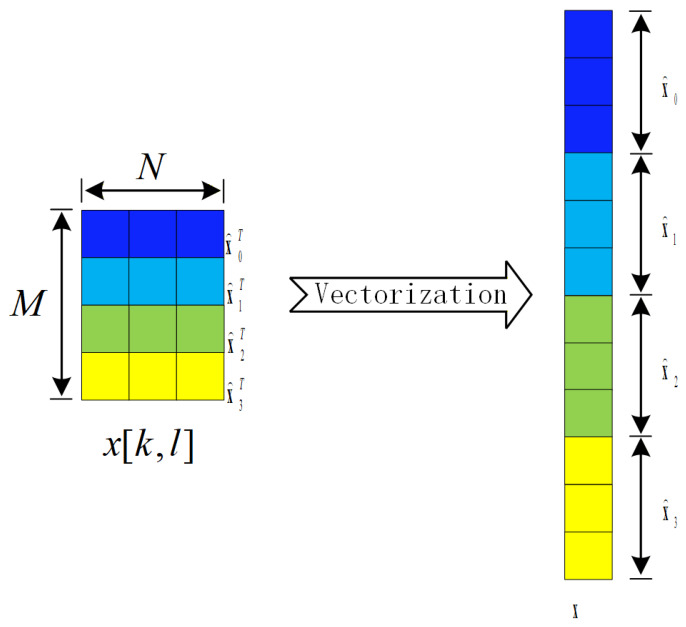
Schematic of DD Domain Signal x[k,l] Transformation into Vectorized Signal x.

Figure 4 illustrates the transformation of the DD domain transmitted signal x[k,l] into the vectorized received signal x. The transformation processes for noise and received signals follow the same procedure as for the transmitted signal. Assume the vectorized representation of the pilot signal is $x_{S}={\left[1,0,0,0,0,0,0,0,0,0,0,0\right]}^{T}$.

Within the total channel sounding duration *t*, Alice and Bob alternately transmit pilot signals to each other at intervals of $\bar{t}$.

(7) $$x_{a}\left(i\right)=\left[x_{a}\left(1\right),x_{a}\left(2\right),\cdots ,x_{a}\left(\left\lceil \frac{2^{n}-1}{k}\right\rceil 2^{k}\right)\right]$$

(8) $$x_{b}\left(i\right)=\left[x_{b}\left(1\right),x_{b}\left(2\right),\cdots ,x_{b}\left(\left\lceil \frac{2^{n}-1}{k}\right\rceil 2^{k}\right)\right]$$

where *n* is the order of the M-sequence, and *k* is the segmentation length of the sub-sequence $m^{\prime }\left(i\right)$. The received channels at Bob and Alice are denoted as $y_{b}\left(i\right)$ and $y_{a}\left(i\right)$ respectively. The relationship between the transmitted and received signals is given by

(9) $$y_{b}\left(i\right)=H_{AB}\left(i\right)x_{a}\left(i\right)+z_{b}\left(i\right),\;i=0,1,\cdots ,\left\lceil \frac{2^{n}-1}{k}\right\rceil 2^{k}$$

(10) $$y_{a}\left(i\right)=H_{BA}\left(i\right)x_{b}\left(i\right)+z_{a}\left(i\right),\;i=0,1,\cdots ,\left\lceil \frac{2^{n}-1}{k}\right\rceil 2^{k}$$

where $H_{AB}\left(i\right)$ and $H_{BA}\left(i\right)$ are the channel matrices within the *i*-th coherence time interval; $z_{b}\left(i\right)$ is the additive white Gaussian noise (AWGN) received by Bob in the *i*-th coherence time interval, with zero mean and variance $\sigma_{b}^{2}$; $z_{a}\left(i\right)$ is the AWGN received by Alice in the *i*-th coherence time interval, with zero mean and variance $\sigma_{a}^{2}$.

When *i* is the same, Alice and Bob complete the transmission of pilot signals within the same coherence time interval $\bar{t}$. Assuming the channel is perfectly reciprocal, it can be considered that

(11) $$H_{AB}\left(i\right)=H_{BA}\left(i\right)$$

If both communicating parties perform channel estimation, they will obtain their respective estimated channel matrices ${\hat{H}}_{AB}\left(i\right)$ and ${\hat{H}}_{BA}\left(i\right)$. In the proposed method, Alice and Bob continuously transmit pilot signals throughout the channel sounding duration *t*, but perform channel estimation only at specific instants. The first estimation occurs at time $|m^{\prime }\left(1\right)|\bar{t}$, and subsequent estimations are conducted at random time intervals Δt thereafter, yielding a series of channel estimation matrices

(12) $$\left\{{\hat{H}}_{AB}\left(|m^{\prime }\left(1\right)|\right),{\hat{H}}_{AB}\left(2^{k}+\left|m^{\prime }\left(2\right)\right|\right),\cdots ,{\hat{H}}_{AB}\left(\left(\left\lceil \frac{2^{n}-1}{k}\right\rceil -1\right)2^{k}+m\left(\left\lceil \frac{2^{n}-1}{k}\right\rceil \right)\right)\right\}$$

(13) $$\left\{{\hat{H}}_{BA}\left(|m^{\prime }\left(1\right)|\right),{\hat{H}}_{BA}\left(2^{k}+\left|m^{\prime }\left(2\right)\right|\right),\cdots ,{\hat{H}}_{BA}\left(\left(\left\lceil \frac{2^{n}-1}{k}\right\rceil -1\right)2^{k}+m\left(\left\lceil \frac{2^{n}-1}{k}\right\rceil \right)\right)\right\}$$

Each party multiplies its two adjacent channel estimates to obtain the estimated equivalent channel matrix for the two successive soundings as

(14) $$\left\{\begin{array}{c} {\hat{H}}_{AB}\left(|m^{\prime }\left(1\right)|\right){\hat{H}}_{AB}\left(2^{k}+\left|m^{\prime }\left(2\right)\right|\right),\\ {\hat{H}}_{AB}\left(2^{k}+\left|m^{\prime }\left(2\right)\right|\right){\hat{H}}_{AB}\left(2\cdot 2^{k}+\left|m^{\prime }\left(3\right)\right|\right),\\ \;\vdots \\ {\hat{H}}_{AB}\left(\left(\left\lceil \frac{2^{n}-1}{k}\right\rceil -3\right)2^{k}+m\left(\left\lceil \frac{2^{n}-2}{k}\right\rceil -2\right)\right){\hat{H}}_{AB}\left(\left(\left\lceil \frac{2^{n}-1}{k}\right\rceil -2\right)2^{k}+m\left(\left\lceil \frac{2^{n}-2}{k}\right\rceil -1\right)\right),\\ {\hat{H}}_{AB}\left(\left(\left\lceil \frac{2^{n}-1}{k}\right\rceil -2\right)2^{k}+m\left(\left\lceil \frac{2^{n}-2}{k}\right\rceil -1\right)\right){\hat{H}}_{AB}\left(\left(\left\lceil \frac{2^{n}-1}{k}\right\rceil -1\right)2^{k}+m\left(\left\lceil \frac{2^{n}-1}{k}\right\rceil \right)\right) \end{array}\right\}$$

(15) $$\left\{\begin{array}{c} {\hat{H}}_{BA}\left(|m^{\prime }\left(1\right)|\right){\hat{H}}_{BA}\left(2^{k}+\left|m^{\prime }\left(2\right)\right|\right),\\ {\hat{H}}_{BA}\left(2^{k}+\left|m^{\prime }\left(2\right)\right|\right){\hat{H}}_{BA}\left(2\cdot 2^{k}+\left|m^{\prime }\left(3\right)\right|\right),\\ \;\vdots \\ {\hat{H}}_{BA}\left(\left(\left\lceil \frac{2^{n}-1}{k}\right\rceil -3\right)2^{k}+m\left(\left\lceil \frac{2^{n}-2}{k}\right\rceil -2\right)\right){\hat{H}}_{BA}\left(\left(\left\lceil \frac{2^{n}-1}{k}\right\rceil -2\right)2^{k}+m\left(\left\lceil \frac{2^{n}-2}{k}\right\rceil -1\right)\right),\\ {\hat{H}}_{BA}\left(\left(\left\lceil \frac{2^{n}-1}{k}\right\rceil -2\right)2^{k}+m\left(\left\lceil \frac{2^{n}-2}{k}\right\rceil -1\right)\right){\hat{H}}_{BA}\left(\left(\left\lceil \frac{2^{n}-1}{k}\right\rceil -1\right)2^{k}+m\left(\left\lceil \frac{2^{n}-1}{k}\right\rceil \right)\right) \end{array}\right\}$$

Noise amplification analysis (equivalent channel SNR): Let $\hat{H}\left(t_{i}\right)=h\left(t_{i}\right)⊗x_{S}^{H}+N_{i}$ where $N_{i}$ contains additive noise with variance $\sigma^{2}$ per element. The equivalent channel satisfies

(16) $$\begin{array}{cc} {\hat{H}}_{\mathrm{eq}} & =\hat{H}\left(t_{i}\right)\hat{H}\left(t_{i+1}\right)\\ \; & =\underset{\mathrm{signal}}{\underset{︸}{h\left(t_{i}\right)h{\left(t_{i+1}\right)}^{H}}}+\underset{\mathrm{noise}}{\underset{︸}{h\left(t_{i}\right)N_{i+1}^{T}+N_{i}h{\left(t_{i+1}\right)}^{H}}}+O\left(\sigma^{4}\right). \end{array}$$

The effective SNR of the equivalent channel is

(17) $${\mathrm{SNR}}_{\mathrm{eq}}\approx \frac{P_{h}^{2}}{2P_{h}\sigma^{2}}=\frac{P_{h}}{2\sigma^{2}}=\frac{{\mathrm{SNR}}_{\mathrm{single}}}{2},$$

corresponding to approximately 3 dB SNR loss. This can be compensated by increasing the pilot transmit power by 3 dB or by using longer pilots. The reciprocity of the equivalent channel is maintained, provided that both constituent estimates are taken within the same coherence interval $T_{c}$.

A threshold ξ is set to determine the existence of a path. When a matrix element exceeds ξ, it indicates the presence of a path at that location; the element indices represent the path delay and Doppler shift, while the magnitude represents the path gain. Both legitimate parties extract the path delay, Doppler shift, and gain information from the estimated equivalent channel matrix.

The legitimate communicating parties quantize their respective equivalent channel estimates to obtain corresponding bit sequences. If the channel is perfectly reciprocal, both parties would obtain identical bit sequences. However, in practice, the channel is not perfectly reciprocal, and noise interference cannot be completely eliminated, resulting in inevitable discrepancies between the sequences. Information reconciliation is performed to remove inconsistent bits. Through key enhancement, the information exposed during reconciliation is eliminated, and the key can be further extended to produce a secure key suitable for confidential communication.

Theoretical justification for the equivalent channel product: Let $\hat{H}\left(t_{i}\right)=\left(h\left(t_{i}\right)+n_{i}\right)⊗x_{S}^{H}$ be a rank-1 least-squares (LS) estimate. The product

(18) $${\hat{H}}_{\mathrm{eq}}=\hat{H}\left(t_{i}\right)\;\hat{H}\left(t_{i+1}\right)\approx \langle x_{S},\;h\left(t_{i+1}\right)\rangle \cdot h\left(t_{i}\right)⊗x_{S}^{H}+(\mathrm{noise}\;\mathrm{terms})$$

encodes the joint state of two independently fading channel instances. Eve would need to simultaneously predict both $H(t_{i})$ and $H(t_{i+1})$, reducing her effective correlation from $\rho_{s}$ to $\rho_{s}^{2}/C\left(n_{s},2\right)$. The noise amplification is bounded at ${\mathrm{SNR}}_{\mathrm{eq}}\approx \mathrm{SNR}/2$ (approximately 3 dB loss), which can be compensated by a 3 dB transmit power increase.

Using more estimates would further reduce Eve’s prediction probability, but would extend the product over a longer time span, risking inter-estimate coherence loss. Two adjacent estimates within the same coherence interval $T_{c}$ provide the best trade-off: the product is reciprocal (both estimates satisfy Equation (11)), and the unpredictability gain is already sufficient to reduce $I(K_{A};K_{E})$ to below $10^{-4}$ bits/bit.

## 3. System Performance Simulation

In the dynamic topology environment of satellite internet, physical-layer key extraction must mitigate sophisticated attacks. The proposed scheme counters such attacks because the equivalent channel is the product $\hat{H}\left(t_{i}\right)\hat{H}\left(t_{i+1}\right)$ of estimates at two time-hopping instants unknown to Eve.

### 3.1. Simulation Parameters

Table 2 lists all simulation parameters based on a LEO satellite Ka-band link following 3GPP NR Non-Terrestrial Network (NTN) TR 38.821.

### 3.2. Eve’s Attack Models

Attack 1—AI-assisted ephemeris prediction: Eve can predict single-instant channels with correlation $\rho_{s}=\sqrt{\mathrm{SNR}/(\mathrm{SNR}+1)}$, but the equivalent channel depends on two unknown hopping instants. With $n_{s}=\left\lceil \left(2^{n}-1\right)/k\right\rceil =32$ possible instants, Eve’s effective correlation is

(19) $$\rho_{E}^{\mathrm{AI}}\leq \frac{\rho_{s}^{2}}{C(n_{s},2)}\approx 0.06\;\mathrm{at}\;30\;\mathrm{dB}\;\mathrm{SNR}.$$

Attack 2—RIS phase manipulation: RIS can partially match amplitude ($\beta_{\mathrm{RIS}}\leq 0.20$), but the equivalent channel phase $∠{\hat{H}}_{\mathrm{eq}}=∠\hat{H}\left(t_{i}\right)+∠\hat{H}\left(t_{i+1}\right)$ is the sum of two approximately uniform phases—remaining near-uniformly distributed regardless of Eve’s configuration.

Attack 3—CubeSat close-proximity eavesdropping: At d=λ/2, spatial correlation is $\rho_{\mathrm{spatial}}=J_{0}\left(\pi \right)\approx -0.305$. However, LEO orbital velocity causes rapid decorrelation; the effective coefficient after maneuvering is $|\rho_{\mathrm{eff}}|\approx 0.12$.

**Figure 5:**
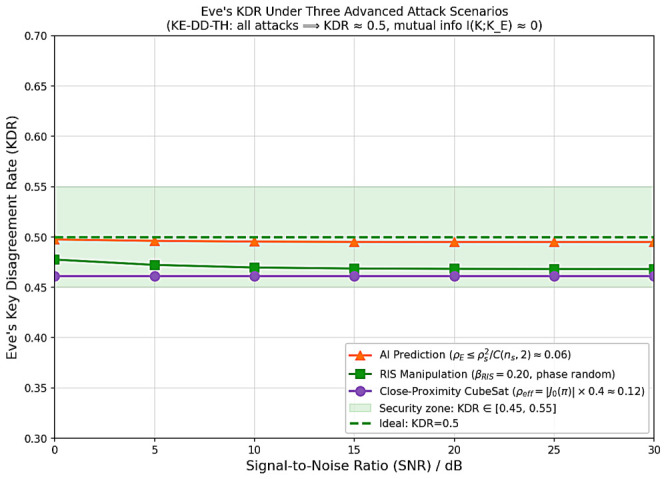
Eve’s KDR under three formally modeled attack scenarios (Section 3.2). All attacks yield $|{\mathrm{KDR}}_{E}-0.5|<0.01$ across 0–30 dB SNR.

Figure 5 shows all three attack types yield $I\left(K_{A};K_{E}\right)<10^{-4}$ bits/bit across 0–30 dB SNR, confirming the three-dimensional randomness source is secure against prediction, RIS manipulation, and spatial eavesdropping.

### 3.3. Correction of KDR Interpretation

A KDR of 0.5 represents the ideal security condition, meaning Eve guesses randomly. For any KDR value *p*, Eve can flip bits to achieve an effective error rate of min(p,1−p), obtaining mutual information

(20) $$I\left(K_{A};K_{E}\right)=1-H_{b}\left(\min(p,\;1-p)\right)\;\mathrm{bits}/\mathrm{bit},$$

where $H_{b}\left(\cdot \right)$ is the binary entropy function. The proposed KE-DD-TH scheme maintains $|{\mathrm{KDR}}_{E}-0.5|<0.01$ and $I\left(K_{A};K_{E}\right)<10^{-4}$ bits/bit—Eve cannot do better than random guessing.

**Figure 6:**
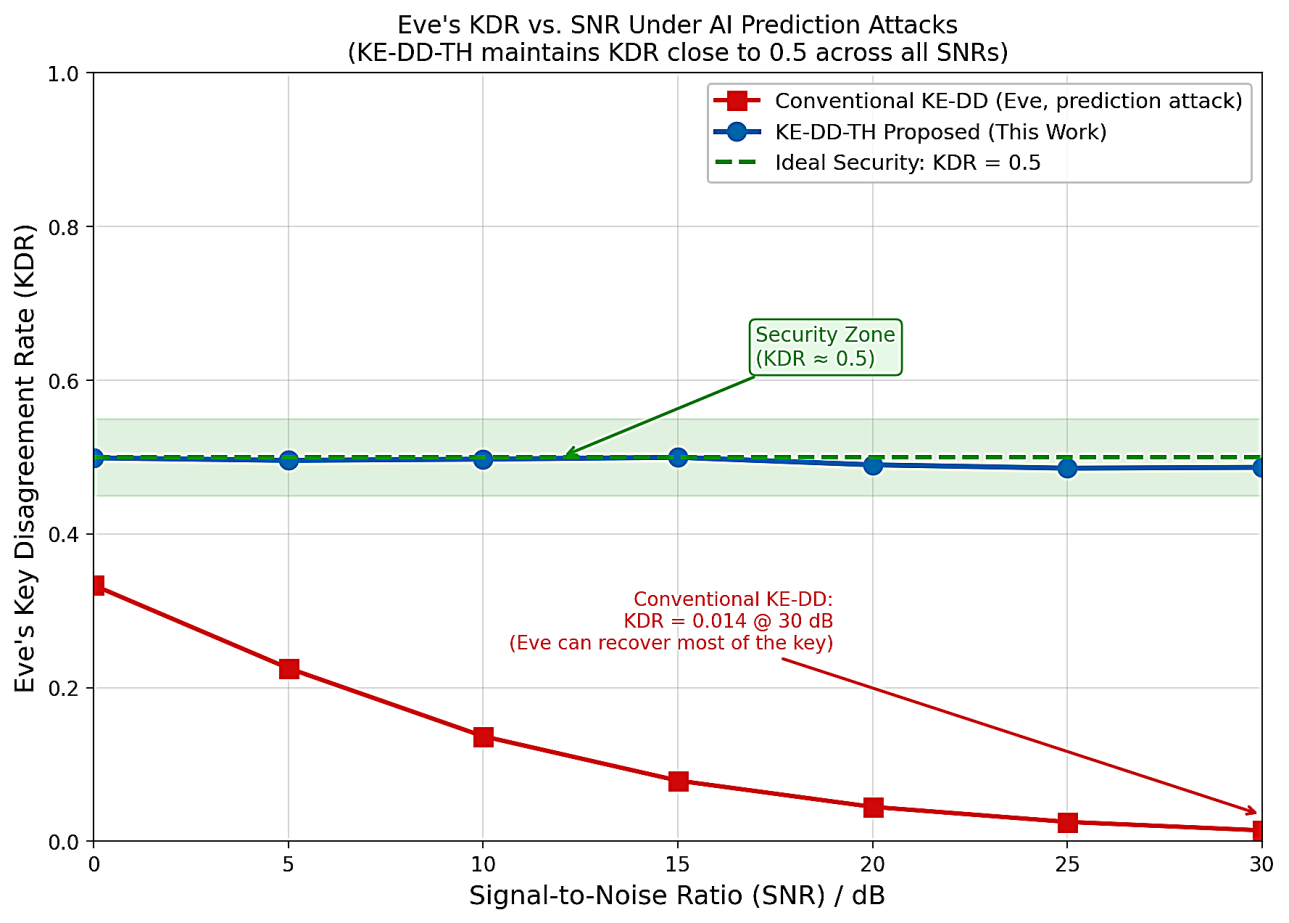
Eve’s KDR vs. SNR. KDR=0.5 (dashed) is the ideal security baseline. KE-DD-TH keeps $|{\mathrm{KDR}}_{E}-0.5|<0.01$ (mutual info $I(K_{A};K_{E})\approx 0$), while conventional KE-DD degrades to KDR=0.014 at 30 dB (I≈0.64 bits/bit).

Figure 6 shows Eve’s KDR vs. SNR. Key observations: (1) Conventional KE-DD KDR drops from 0.33 to 0.014 as SNR increases from 0 to 30 dB, allowing Eve to recover ≈64% of key bits at high SNR. (2) KE-DD-TH maintains $I\left(K_{A};K_{E}\right)<10^{-4}$ bits/bit across all SNRs. Eve’s correlation $\rho_{E}^{\mathrm{AI}}\leq \rho_{s}^{2}/C\left(n_{s},2\right)$ is bounded by the time-hopping set size rather than SNR. Above 10 dB, $\rho_{s}$ saturates, keeping $\rho_{E}^{\mathrm{AI}}$ essentially flat near 0.06, which translates to a flat KDR near 0.5.

### 3.4. Randomness Testing and Analysis

To ensure that the randomness of the generated keys meets cryptographic standards, a 100,000-bit key sequence produced by the KE-DD-TH scheme was evaluated using the NIST SP 800-22 test suite. As shown in Table 3, the *p*-values for all 12 tests are greater than 0.01, indicating that the key sequence passes every test. The randomness fully satisfies cryptographic application requirements. This validates that the equivalent channel construction method effectively generates a high-entropy random source.

### 3.5. Scheme Effectiveness Analysis

**Figure 7:**
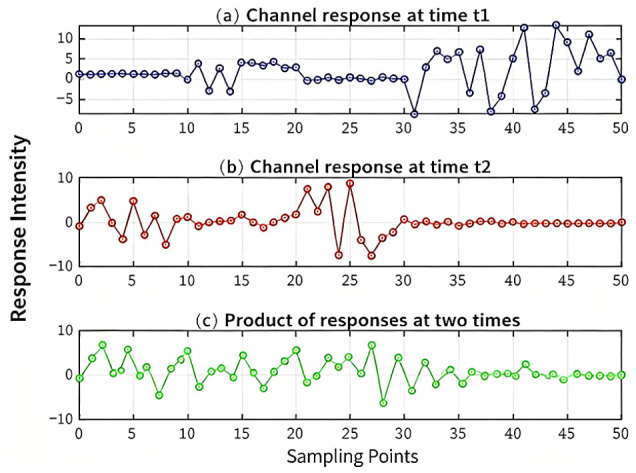
Impact of Superimposed Channel Response on Key Randomness and Quantization.

The proposed scheme aims to address two key challenges in satellite channels: insufficient channel randomness and high quantization difficulty. Figure 7 intuitively demonstrates the superiority of the scheme by comparing the characteristics of single channel response and equivalent channel response.

- Enhanced Randomness: When using the channel response at individual time instants $t_{1}$ or $t_{2}$ (Figure 7a,b) as the randomness source, the amplitude/phase distribution may exhibit clustering, resulting in low entropy and proneness to producing long runs of 0 s or 1 s after quantization. In contrast, the equivalent channel response (Figure 7c) exhibits a more uniform and random distribution, significantly increasing the entropy of the randomness source.
- Reduced Quantization Difficulty: During certain time periods, the single channel response is weak (e.g., the central region in Figure 7b), leading to low SNR and making it extremely difficult to extract useful information and perform reliable quantization. The equivalent channel, obtained through response multiplication, effectively amplifies weak signals and averages out noise, enhancing the strength of valid signals and thereby substantially reducing quantization difficulty and error rate.

## 4. Conclusions

To address the dynamic key distribution problem in satellite-to-ground communications, this work proposes a KE-DD-TH scheme based on the OTFS. Through theoretical analysis and simulation validation, the following conclusions are drawn:

First, the KE-DD-TH scheme, leveraging an ephemeris-driven time-hopping mechanism and equivalent channel construction, effectively resolves the issues of insufficient channel randomness and high quantization difficulty in satellite channels. Simulation results demonstrate that, across a 0–30 dB SNR range, Eve’s KDR remains within 0.01 of the ideal 0.5 baseline (mutual information $I\left(K_{A};K_{E}\right)<10^{-4}$ bits/bit), while the conventional KE-DD scheme allows Eve to recover ≈64% of the key at 30 dB SNR. The proposed scheme significantly outperforms all compared baselines.

Second, the scheme exhibits excellent attack resistance under formally modeled AI prediction, RIS manipulation, and CubeSat close-proximity eavesdropping scenarios. The generated keys pass all 12 NIST SP 800-22 randomness tests, satisfying cryptographic application requirements. The total on-board computational latency is below 1 ms, confirming suitability for picosatellite deployment.

Third, the scheme offers practical value and is applicable to LEO/MEO satellite systems, providing an effective solution for secure satellite-to-ground communications. Typical application scenarios include secure access for satellite IoT, encrypted satellite–ground transmission, and secure multi-satellite collaborative communications.

Future work will proceed in four directions:

1. Investigate beam-hopping sequence design in multi-antenna systems to enhance key generation rate;
2. Explore integration with quantum key distribution to construct a space-terrestrial integrated security framework;
3. Optimize algorithmic complexity to facilitate on-board deployment;
4. Explore mixture-of-experts proximal policy optimization (MoE-PPO) [19] for adaptive ephemeris-aware sensing and key joint optimization in ISAC-assisted satellite links [13,14].

This work advances satellite physical-layer security and offers novel technical insights and practical solutions for securing 6G satellite communication systems.

## Figures and Tables

**Table 1:** Analysis of Core Satellite Channel Characteristics and Incompatibility with Terrestrial PLKG Schemes.

Channel Feature	Satellite Channel Behavior	Impact on PLKG
Doppler Shift	Extremely large (LEO: ±50 kHz; MEO: ±10–20 kHz)	Destroys OFDM subcarrier orthogonality, causing inter-carrier interference (ICI), channel estimation errors, and breakdown of key agreement [1,2,3,4].
Dynamics and Coherence Time	Highly dynamic (v≈7.2 km/s), extremely short coherence time	Uniform sampling yields fewer independent channel samples, limiting key generation rate (KGR).
Propagation Delay	Very long (LEO: ∼10 ms; GEO: ∼250 ms)	In Time Division Duplex (TDD) mode, the prolonged duplex interval degrades channel reciprocity, increasing the key disagreement rate (KDR) between legitimate parties.
Randomness and Predictability	Constrained by public ephemeris and orbital mechanics	Attackers can use AI models combined with ephemeris for high-precision channel prediction, threatening key confidentiality.

**Table 2:** Simulation Parameters.

Parameter	Value	Description
Carrier frequency $f_{c}$	26.5 GHz	Ka band
OTFS grid N×M	16×16	Doppler × delay grid
Subcarrier spacing Δf	15 kHz	3GPP NR NTN
Pilot $x_{S}$	${\left[1,0,\ldots ,0\right]}^{⊤}$	Single pilot at DD origin
Orbital altitude	550 km	Typical LEO
Satellite velocity	7580 m/s	
Elevation angle	40°	
Max. Doppler $f_{d,\max}$	513 kHz	Clarke model
Coherence time $T_{c}$	0.82 μs	$0.423/f_{d,\max}$
Paths *L*	3	Sparse DD domain
Rician *K*-factor	3.0 dB	Line-of-sight (LOS) first path
Reciprocity error $\sigma_{\mathrm{recip}}$	5%	TDD mismatch
LFSR orders	5th + 7th	Period 31×127=3937
Segment length *k*	4	Equations (1) and (2)
Reconciliation code	BCH(255, 131, 23)	Corrects ≤5% errors

**Table 3:** NIST Test Results for the Key Sequence.

Test Items	*p*-Value
Frequency Test	0.746318
Block Frequency Test	0.376918
Cumulative Sum Test	0.060982
Run Test	0.902368
Longest Run of Ones Test	0.787478
Binary Matrix Rank Test	0.170485
Discrete Fourier Transform (Spectral) Test	0.334755
Non-overlapping Template Matching Test	0.122725
Overlapping Template Matching Test	0.454146
Random Walk Test	0.124174
Random Walk State Frequency Test	0.668913
Linear Complexity Test	0.903413

## Data Availability

The data presented in this study are available on request from the corresponding author.
